# Antagonism of the histamine H_4_ receptor reduces LPS-induced TNF production in vivo

**DOI:** 10.1007/s00011-013-0612-5

**Published:** 2013-03-27

**Authors:** Jeffery M. Cowden, Fuqu Yu, Mamatha Challapalli, Jing-Feng Huang, Sunhwa Kim, Wai-Ping Fung-Leung, Jing Ying Ma, Jason P. Riley, Mai Zhang, Paul J. Dunford, Robin L. Thurmond

**Affiliations:** 1 Janssen Research & Development, L.L.C., 3210 Merryfield Row, San Diego, CA 92121 USA; 2 La Jolla BioConsulting, 4653 Carmel Mountain Road Suite 308-245, San Diego, CA 92130 USA; 3 Sialix, Inc., 1396 Poinsettia Ave, Vista, CA 92081 USA

**Keywords:** Histamine, Macrophages, Kupffer cells, Toll-like receptors

## Abstract

**Objective:**

Antagonism of the histamine H_4_ receptor (H_4_R) has been shown to be anti-inflammatory in a number of preclinical disease models, however the exact mechanisms behind this are still being uncovered. In vitro, the receptor interacts with TLR and impacts inflammatory mediator production from a number of different cell types. Here it is shown that this interaction also occurs in vivo.

**Materials and methods:**

Wild-type and H_4_R deficient BALB/c mice received an i.p. injection of LPS in PBS in conjunction with p.o. JNJ 7777120 or JNJ 28307474 (H_4_R antagonists). Two hours later blood was collected and TNF was measured.

**Results:**

Two different H_4_R antagonists inhibited LPS-induced TNF production in mice and this production was also reduced in H_4_R-deficient mice. The TNF mRNA analysis showed that the major source of the cytokine was the liver and not blood, and that the H_4_R antagonist only reduced the expression levels in the liver. Depletion or inactivation of macrophages reduced the TNF levels and eliminated the H_4_R sensitivity. Treatment with an H_4_R antagonist also reduced LPS-induced liver injury and blocked LPS-enhanced lung inflammation in mice.

**Conclusion:**

The data support an interaction between H_4_R and TLR activation in vivo that can drive inflammatory responses.

**Electronic supplementary material:**

The online version of this article (doi:10.1007/s00011-013-0612-5) contains supplementary material, which is available to authorized users.

## Introduction

The histamine H_4_ receptor (H_4_R) has been implicated in a variety of pathophysiological functions due to effects seen in preclinical models [1]. Most of the data have involved effects on inflammatory processes. H_4_R antagonists reduced inflammation in mouse models of asthma [2, 3] and dermatitis [4] and similar effects were observed with H_4_R-deficient mice [2, 4]. However, the exact molecular mechanisms are not clear. The H_4_R has been shown to mediate chemotaxis of cells such as eosinophils [5, 6], mast cells [7, 8] and dendritic cells [9, 10] and this may explain some of its role in inflammation. In addition, the receptor has been shown to modulate inflammatory mediator production in vitro from dendritic cells, mast cells, T cells and monocytes [2, 11–13]. In most cases, these findings have been in the context of TLR stimulation and it is intriguing to speculate that there may be an interaction between inflammatory signals that activate TLR pathways and those that activate the H_4_R. Indeed, in mouse mast cells it has been shown that the H_4_R can potentiate LPS-induced IL-6 production and this interaction occurs at the level of kinase activation [11].

Here the role of the H_4_R in TLR-driven inflammation is explored in vivo. The LPS-induced TNF production in vivo is inhibited in H_4_R-deficient mice or by treatment with H_4_R antagonists. The interaction between the H_4_R and TLR provides further mechanistic support for the role of the H_4_R in inflammation.

## Materials and methods

### Materials

Wild-type BALB/c mice were obtained from Charles River Laboratories (Wilmington, MA). H_4_R deficient mice were generated by Lexicon Genetics (Woodlands Park, TX) as previously described [7]. Mice were housed in community cages on a 12-h light cycle and fed mouse chow and water ad libitum. All procedures were performed according to the internationally accepted guidelines for the care and use of laboratory animals in research and were approved by the local Institutional Animal Care and Use Committee. The JNJ 7777120 and JNJ 28307474 (H_4_R antagonists) were synthesized as previously described [4, 14]. In all cases H_4_R antagonists were delivered p.o. in 20 % hydroxy-propyl-β-cyclodextran. Lipopolysaccharide (E.coli 0111:B4), methylpalmitate (MP; stock 200 mg/ml in 5 % glucose solution containing 0.2 % Tween-20) and galactosamine (GaIN) were purchased from Sigma-Aldrich, LLC, (St. Louis, MO). Clodronate liposomes (CL) were purchased from ClodronateLiposomes.org (Amsterdam, Netherlands) in a stock solution of around 5 mg/ml in PBS. The stock was further diluted in PBS to 1 mg/ml just before administration and tail vein injection at ~200 μl/mouse. The TNF ELISA was obtained from R&D Systems, Inc. (Minneapolis, MN). Low endotoxin RPMI media was from Sigma-Aldrich Co. LLC. (St. Louis, MO).

### In vivo LPS model

Female wild-type or H_4_R-deficient mice were administered vehicle, JNJ 7777120 or JNJ 28307474 p.o. Thirty minutes later, the mice received an i.p. injection of 20 μg of LPS in PBS. The dose of LPS was selected based on pilot experiments and is consistent with previous reports [15]. Two hours later blood was collected and serum cytokines were measured via ELISA. To assess the activation of NFκB and AP-1, nuclear and cytoplasmic fractions of whole liver tissue sections were prepared using Nuclear Extraction Kit (Active Motif, Carlsbad, CA) with the manufacturer’s protocol. Five μg of proteins from each fraction of individual samples were analyzed for the phosphorylation and/or nuclear translocation of NFκB and AP-1 transcription factors utilizing TransAM NFκB Family Kit (Active Motif, Carlsbad, CA) and TransAM AP-1 Family Kit (Active Motif, Carlsbad, CA) according to the manufacturer’s protocol.

### Kupffer cell depletion

For Kupffer cell depletion, the mice were treated by tail vein injection with either vehicle, 2 g/kg MP or 10 mg/kg CL (the vehicle for CL was empty liposomes in PBS). Forty-four hours after treatment with MP or CL 20 μg of LPS in PBS was administered by i.p. Two hours post LPS injection, animals were euthanized and blood collected via cardiac puncture. Serum TNF levels were quantitated by ELISA. Livers were collected in RNAlater reagent (Ambion, Inc., Austin, TX) and total RNA was extracted using RNeasy Plus Mini Kit (QIAGEN, Inc., Valencia, CA). In addition, blood RNA was extracted using QIAamp RNA Blood Mini Kit (QIAGEN, Inc. Valencia, CA). The RNA was reverse-transcribed to cDNA using the GeneAmp Gold RNA PCR Core Kit (Applied Biosystems, Carlsbad, CA). The TNF message levels were detected using commercially available TaqMan primer/probe sets (Applied Biosystems, Carlsbad, CA) with 7500 Real Time PCR System (Applied Biosystems, Carlsbad, CA). The data is presented as the fold-induction compared to naïve animals.

To assess macrophage depletion, liver samples were fixed in 10 % formalin for 24 h, processed, paraffin embedded, sectioned at 5 μm thickness and mounted on the slides. The slides were deparaffinized and hydrated in PBS followed by blocking the endogenous peroxide with 3 % hydrogen peroxide. Incubation with a solution of 3 % trypsin was used to enhance the binding of the primary antibody. Avidin and biotin blocks (Vector laboratories, Burlingame, CA) were used to avoid nonspecific labeling with primary antibody. To avoid nonspecific reaction with the secondary antibody, slides were pretreated with 10 % normal goat serum before incubation with primary antibody. Rat anti-mouse F4/80 at a concentration of 1:50 (AbD Serotec, Raleigh, NC) as primary antibody and goat anti-rat biotinlated IgG as secondary antibody (Cat#AP183b, Chemicon International, Inc., Temecula, CA) at 1:2,000 concentration were used. Normal rat IgG at 1:50 concentration (Cat# CBL606, Chemicon International, Inc., Temecula, CA) was used as a negative control. The immunoreactivity was visualized by ABC reagent (Vector Laboratories, Burlingame, CA) and diaminobenzidine (DAKO, Carpinteria, CA) followed by counterstaining with Mayer’s hematoxylin. A Nikon E800 light microscope equipped with a Q Image camera was used to capture images. Images were measured by Image Pro Plus software 7.0 (MediaCybernetics, Inc., Bethesda, MD). Positively stained, centro-lobularly located macrophages (Kupffer cells) were automatically counted in the randomly chosen five hot fields under the ×100 magnification objective lens using the cell-count feature of Image Pro Plus 7.0 software. The mean value of the cell counts in five fields was calculated, defined as cell count per field.

### In vitro and ex vivo LPS stimulation of whole blood

For in vitro stimulation, blood from wild-type and H_4_R-deficient mice was diluted 1:1.8 with low endotoxin RPMI media containing vehicle (0.4 % DMSO) or 50 μM JNJ 7777120 and incubated for 1 h at 37 °C. After incubation, 10 ng/ml LPS was added and the samples were incubated for 4 h at 37 °C. Cells were centrifuged, supernatants were harvested and TNF levels were measured by ELISA. For the ex vivo stimulation, wild-type mice were dosed p.o. with JNJ 7777120 (1, 5 and 20 mg/kg). Mice were sacrificed 15 min later (the *T*_max_ for the plasma levels of JNJ 7777120) and blood collected. Blood was diluted 1:2 with low endotoxin RPMI media containing 10 ng/ml LPS and the samples were incubated for 4 h at 37 °C. Cells were centrifuged, supernatants were harvested and TNF levels were measured by ELISA.

### Immunofluorescence for dual F4/80 and TNF staining

Mice received an i.p. injection of 20 μg of LPS (Sigma-Aldrich Co. LLC, St. Louis, MO) or vehicle (PBS) and 2 h later livers, lungs and spleens were harvested and frozen in tissues cassettes with O.C.T. freezing compound. Immunofluorescence detection was carried out by Seventh Wave Laboratories LLC (Chesterfield, MO). Rat anti-mouse F4/80 (monoclonal IgG2b; AbD Serotec) and Dylight 488 mouse anti-rat IgG (green; Thermo Fisher Scientific, Inc. Rockford, IL) was used for F4/80 detection. Rabbit anti-human TNF (polyclonal IgG fraction; Sigma-Aldrich Co. LLC, St. Louis, MO) and Dylight 594 mouse anti-rabbit IgG (red; Thermo Fisher Scientific, Inc. Rockford, IL) was used for TNF detection. The 4′, 6-diamidino-2-phenylindole was used to provide a nuclear counterstain in all the sections. The frozen blocks were sectioned (approximately 6 micrometer thickness) to produce one slide per tissue per mouse, and the sections were immunoreacted to detect TNF and F4/80 Immunofluorescence. A cocktail of rat IgG2b and normal rabbit IgG fraction adjusted to the equivalent protein concentrations as the primary antibodies was substituted for the primary antibody cocktail for the negative control sections.

### H_4_R expression in Kupffer cells

Freshly isolated female mouse and human Kupffer cells were purchased from Celsis In Vitro Technologies (Baltimore, MD). Total RNA was extracted using RNeasy Plus Mini Kit from QIAGEN, Inc. (Valencia, CA). The H_4_R message levels were detected using commercially available TaqMan primer/probe sets (Applied Biosystems, Carlsbad, CA) with 7500 Real Time PCR System (Applied Biosystems, Carlsbad, CA).

### Liver injury model

Female mice were treated with 10 mg/kg LPS + 1 g/kg GaIN in PBS through tail vein injection. One hour before LPS + GaIN treatment, the mice were pretreated with vehicle or 20 mg/kg JNJ 28307474 p.o. Six hours after LPS + GaIN injection, serum was harvested for alanine transaminase (ALT) measurement using ACE Alera system (Alfa Wassermann, West Caldwell, NJ).

### Mouse asthma model

A mouse asthma model was run as previously described [2] with the following modifications. Endotoxin was removed from the OVA by phase separation using Triton X-114 as previously described [16]. Thirty minutes prior to OVA aerosol challenge on day 21 through day 24, animals under light anesthesia were given a 50 μl intranasal dose of PBS or PBS + 1 ng LPS. The JNJ 7777120 was given at 20 mg/kg p.o. 30 min prior to LPS.

## Results

**Fig. 1:**
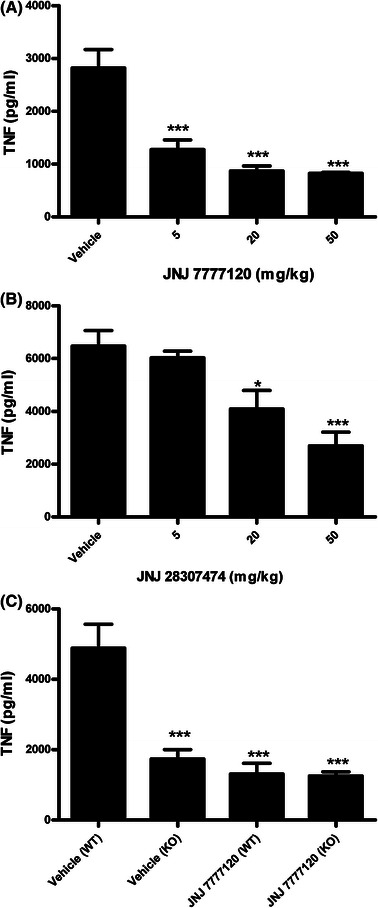
H_4_R antagonists inhibit TNF production in vivo. Wild-type mice (*n* = 5–6 mice per group) were treated orally with either vehicle, JNJ 7777120 (**a**) or JNJ 28307474 (**b**) 30 min prior to i.p. administration of LPS (20 μg). Two hours later the levels of TNF in the serum were analyzed by ELISA. (C) LPS (20 μg) induced serum TNF levels in wild-type (WT) mice but not in H_4_R-deficient mice (KO). In addition, JNJ 7777120 reduced the TNF levels in wild-type mice, but not in the H_4_R-deficient mice. Six to eight mice were used per group. For all panels the average along with SEM is plotted. ***p < 0.001 by one-way ANOVA with post hoc Bonferroni’s test compared vehicle administration in wild-type animals

Previous studies have suggested an interaction between the H_4_R and TLR in vitro [2, 11–13, 17–19]. Based on this, the effects of the H_4_R on LPS-induced cytokine production in vivo was assessed. In mice, TNF production can be detected in serum 2 h after i.p. injection of LPS. When mice were pretreated with the selective H_4_R antagonist JNJ 7777120 30 min before LPS administration, the levels of TNF were dramatically reduced (Fig. 1a). A second structurally distinct antagonist, JNJ 28307474, was also found to inhibit LPS-induced TNF production in a dose dependent fashion, confirming that this effect was due to antagonism of the H_4_R, (Fig. 1b). The difference in dose response between JNJ 7777120 and JNJ 28307474 reflects the weaker affinity of the latter for the mouse H_4_R [4]. In addition to the pharmacological evidence that this is an H_4_R dependent effect, LPS-induced TNF levels were dramatically reduced in H_4_R-deficient mice compared to wild-type mice and, furthermore, JNJ 7777120 at 50 mg/kg had no additional effect in H_4_R-deficient mice (Fig. 1c). The levels of TNF produced show little variability within each group, however these levels do vary across experiments run at different times. The consistent findings in H_4_R-deficient mice and with two structurally distinct antagonists confirm that the suppressive effect on LPS-induced TNF production in vivo is due to antagonism of the H_4_R.

**Fig. 2:**
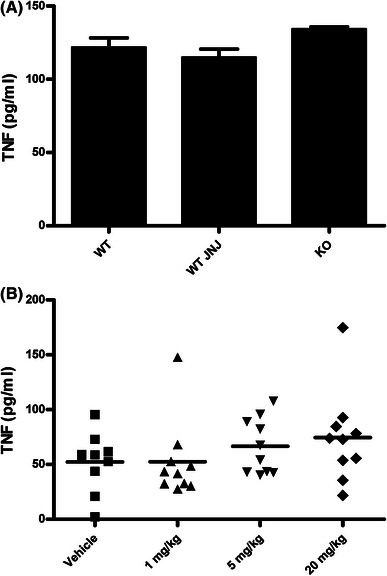
H_4_R antagonists do not inhibit TNF production in vitro or ex vivo. **a** Mouse whole blood was stimulated in vitro with LPS (10 ng/ml) for 4 h and then TNF levels were measured. Blood from BALB/c wild-type mice (WT) and H_4_R-deficient mice (KO) was used. In addition, 50 μM of JNJ 7777120 was added to blood from wild-type mice (WT JNJ) 1 h prior in stimulation with LPS. The analysis was carried out in triplicate and the average along with SEM is plotted. There was no significant difference in the TNF levels between any of the groups. **b** Wild-type mice (9–10 mice per group) were dosed p.o. with vehicle or JNJ 7777120 at the doses given. Fifteen minutes later blood was collected and stimulated with LPS (10 ng/ml) for 4 h before TNF levels were measured. There was no significant difference in TNF levels between any of the groups

Monocytes and macrophages are the most likely source of TNF in this model and, therefore, the effects of H_4_R inhibition on TNF production in whole blood in vitro were explored. Blood from mice was pretreated with JNJ 7777120 in vitro before LPS stimulation. The TNF was produced but no inhibition by JNJ 7777120 was detected (Fig. 2a). The same result was observed with blood from H_4_R-deficient mice compared to wild-type mice (Fig. 2a). This was also the case for ex vivo stimulation of whole blood. Animals were administered various doses of JNJ 7777120 and afterwards blood was stimulated with LPS. Once again, there was no inhibition of TNF production (Fig. 2b). Therefore, H_4_R antagonism inhibited LPS-induced TNF levels in vivo, but not when blood was stimulated directly. This suggests that monocytes in the blood were not the source of TNF production by LPS in vivo.

**Fig. 3:**
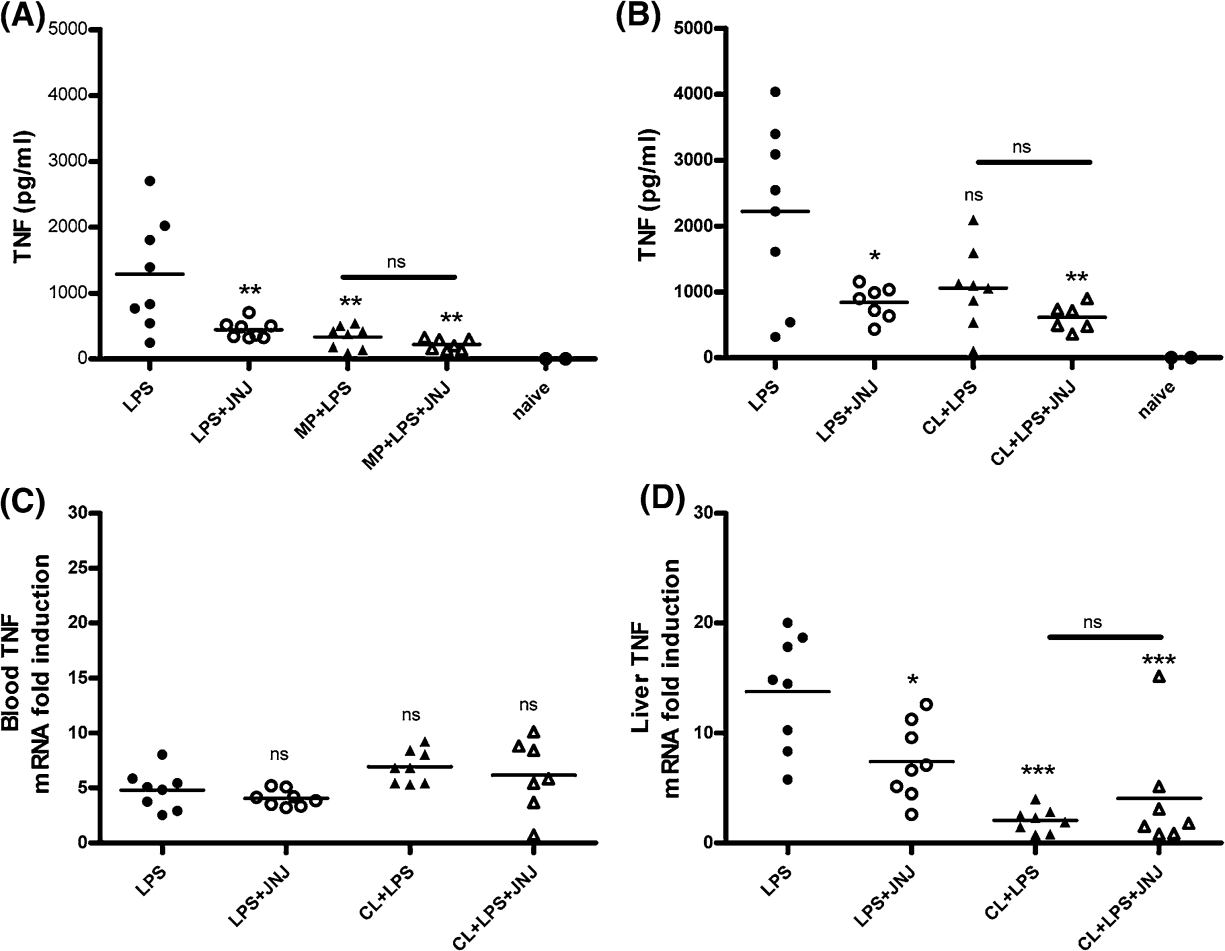
Depletion of liver macrophages reduces LPS-induced TNF levels. Female wild-type mice (7–8 mice per group) were treated with either 20 μg LPS i.p. or LPS i.p. + 20 mg/kg JNJ 7777120 p.o. In addition to this, two groups of mice were treated with vehicle, 2 g/kg MP (**a**) or 10 mg/kg CL (**b**–**d**) 44 h before administration of LPS i.p. The TNF levels were measured in the serum 2 h after LPS stimulation (**a**, **b**). The serum TNF levels were also measured in naïve mice that had no treatment. The TNF mRNA levels were quantitated by RT-PCR in blood (**c**) and in the liver (**d**) from animals treated with 20 μg LPS i.p. ± 20 mg/kg JNJ 7777120 p.o. in the absence or presence of pretreatment with CL. ns, non-significant; ***p < 0.001; **p < 0.01; *p < 0.05 by one-way ANOVA with post hoc Bonferroni’s test compared to LPS alone unless otherwise specified

Tissue resident macrophages are also a potential source of TNF in vivo. In particular, liver resident macrophages are known to respond to inflammatory stimuli. To probe this, methylpalmitate (MP) was used to inactivate tissue resident macrophages. As shown previously, MP does not deplete macrophages (Figure S1) and instead inactivates them [20]. Treatment with MP reduced the LPS-induced TNF production close to baseline levels. Treatment with the H_4_R antagonist had no further effect on the TNF levels (Fig. 3a). To further explore this finding, clodronate liposomes (CL) were used to deplete liver macrophages. Immunohistochemistry staining with F4/80 was used to show that the protocol reduced liver resident macrophage (Figure S2). As with MP, CL treatment reduced the serum levels of TNF to a comparable level as with JNJ 7777120 treatment alone, although in both cases the levels were still slightly higher than the level observed in naïve animals (Fig. 3b). Treatment with JNJ 7777120 after CL depletion of macrophages had no effect on TNF production. This suggests that the source of LPS-induced TNF in vivo may be liver resident macrophages. To confirm this, TNF mRNA levels were measured in the liver and in whole blood after LPS stimulation in vivo. The LPS administration induced a much higher level of TNF mRNA in the liver than in blood (Fig. 3c, d). Treatment with JNJ 77777120 suppressed the TNF mRNA level in the liver, but not in blood (Fig. 3c, d). Furthermore, depletion of tissue macrophages with CL also reduced the TNF mRNA induction in the liver (Fig. 3d).

**Fig. 4:**
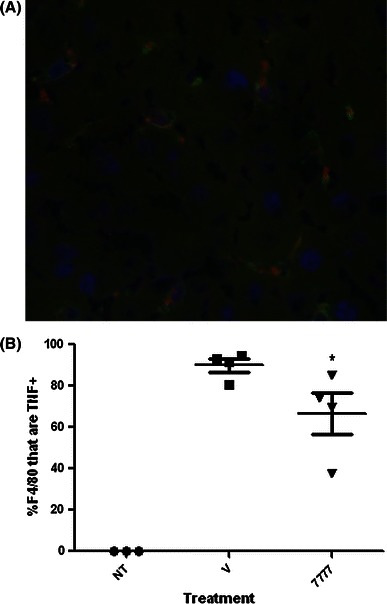
LPS induces TNF production from F4/80^+^ cells in the liver. Livers were collected from untreated mice, mice treated with 20 μg LPS i.p., or mice treated with 20 μg ng/ml LPS i.p. and 20 mg/kg JNJ 7777120. Frozen sections were stained with F4/80 (*green*) and anti-TNF (*red*) and detected by immunofluorescence. A representative image from a mouse treated with LPS is shown in panel **a** and indicates the co-localization of F4/80 and TNF staining. The percentage of F4/80^+^ cells that were TNF^+^ was quantitated from multiple animals in each group (**b**). *p < 0.05 by *t* test comparing the JNJ 7777120 treated and untreated groups (color figure online)

To verify that the LPS-induced TNF is derived from macrophages, immunofluorescence costaining of macrophages and intracellular TNF were conducted after in vivo administration of LPS. In mice not receiving LPS, TNF was not expressed in the liver (Figure S3). In contrast after treatment with LPS almost all F4/80 positive cells in the liver were positive for TNF and all of TNF positive cells were F4/80 positive (Fig. 4a, b). Treatment with the H_4_R antagonist, JNJ 7777120, reduced the percentage of F4/80^+^/TNF^+^ cells (Fig. 4b). Therefore, the data suggests that tissue resident macrophages are the source of LPS-induced TNF in vivo and that antagonism of the H_4_R reduces their activity. This is further supported by the detection of H_4_R mRNA in mouse Kupffer cells albeit at very low levels compared to bone-marrow derived mast cells (data not shown). Human Kupffer cells can also express low levels of the H_4_R, but this was only seen in two of the three donors tested (data not shown). While liver resident macrophages appeared to express TNF upon LPS stimulation, most macrophages in the spleen and lung from wild-type animals did not express TNF with only 11 and 9 % showing expression, respectively.

**Fig. 5:**
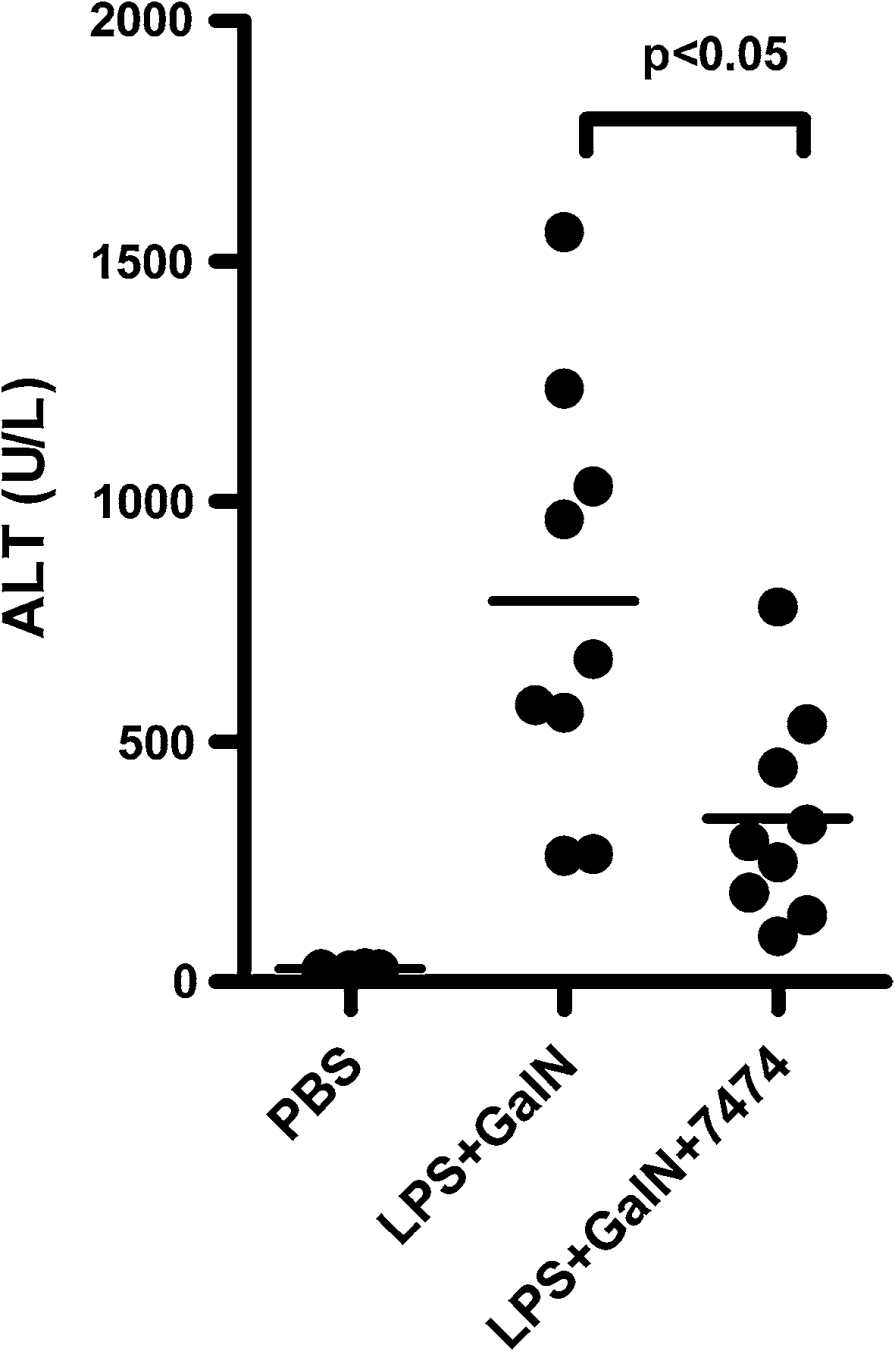
H_4_R antagonism inhibits LPS-induced liver injury. Wild-type mice were pretreated with vehicle (PBS) or JNJ 28307474 before LPS + GaIN injection, and serum ALT levels were measured 6 h later. Statistical significance was determined by one-way ANOVA with post hoc Bonferroni’s test

Suppression of LPS-induced TNF responses in the liver by JNJ 7777120 suggests that antagonizing the H_4_R may help block inflammatory liver injury. The combination of galactosamine (GaIN) and LPS administration in mice leads to increases in ALT indicative of the induction of liver injury and this effect is driven by TNF production [21, 22]. Treatment with the H_4_R antagonist JNJ 28307474 blunts the elevation in ALT suggesting that H_4_R antagonism can inhibit inflammation driven liver injury (Fig. 5). In this case JNJ 28307474 was used instead of JNJ 7777120 since it has a longer half-life in mice and is more appropriate for the time course of the model [Table S1 and 8].

**Fig. 6:**
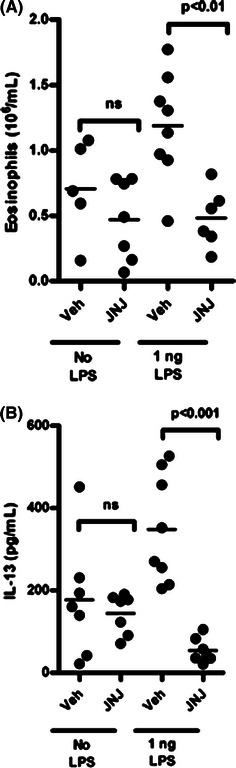
LPS is required for H_4_R-dependent sensitivity in a mouse asthma model. Wild-type mice (*n* = 5–8 per group) were sensitized to ovalbumin i.p. on Days 0 and 14 before repeat aerosol exposure to ovalbumin on Day 21 through 24. The ovalbumin used was either LPS-free (No LPS) or spiked with 1 ng of LPS. Vehicle (Veh) or JNJ 7777120 (JNJ) was administered p.o. 15 min prior to each challenge. **a** The number of eosinophils was calculated from bronchoalveolar lavage fluid collected 24 h after the last OVA challenge. **b** IL-13 levels were measured from bronchoalveolar lavage fluid. Statistical significance was determined by one-way ANOVA with post hoc Bonferroni’s test; *ns* indicated not significant

The data presented here suggest that the H_4_R can mediate LPS responses in the liver and raises the question whether this is reflected in other tissues. Previous work on the H_4_R has shown a role for the receptor in models of asthma indicating that it can mediate lung inflammation. Mouse asthma models are known to be sensitive to the presence of LPS [23, 24]. We exploited this fact to explore the interaction of LPS and the H_4_R in a mouse asthma model. Ovalbumin was first cleaned of any traces of LPS and then used to challenge mice either in the absence or presence of 1 ng of LPS. When the ovalbumin was cleaned of all LPS, the number of eosinophils in the bronchoalveolar lavage fluid was reduced (compare the vehicle groups with and without LPS in Fig. 6a). This effect of low doses of LPS has been described previously [24]. In the absence of LPS, treatment with JNJ 7777120 had no effect on the remaining inflammation. However, when LPS was added back, the number of eosinophils increased and this increase was blocked by the H_4_R antagonist (Fig. 6a). A similar effect was seen when the IL-13 levels from the bronchoalveolar lavage fluid were measured (Fig. 6b). Therefore, the effect of H_4_R antagonism in this mouse model of asthma was dependent on TLR pathways being activated and is consistent with an interaction between TLR and H_4_R activation.

## Discussion

The H_4_R has been suggested to be involved in immune and inflammatory responses and antagonists have shown activity in a number of disease models [1]. However, the exact mechanisms driving these responses have been unclear. There appears to be evidence in vitro of an interaction between inflammation driven by TLR activation and that driven by H_4_R activation. It was reported previously that H_4_R antagonists can inhibit TLR-driven cytokine responses in vitro in mouse dendritic cells and mast cells [2, 11]. In the case of TLR-driven IL-6 production in mast cells, it was suggested that this was due to an interaction in activation of downstream kinases like ERK and phosphoinositide 3-kinase gamma [11]. Therefore, activation of the H_4_R may be very important in amplifying TLR signals. Here we have explored whether a similar interaction occurs in vivo, and whether it could explain some of the in vivo anti-inflammatory effects of H_4_R antagonists.

In this study we have shown that antagonism of the H_4_R can inhibit the in vivo production of TNF induced by LPS. Two different H_4_R antagonists, JNJ 7777120 and JNJ 28307474, have similar effects and inhibition was also seen in H_4_R-deficient mice providing convincing evidence that the effect seen was due to H_4_R antagonism. However, in all cases the level of inhibition was not complete, suggesting that H_4_R-independent factors also play a role.

The inhibition seen with the H_4_R antagonists or in H_4_R-deficient animals only occurs when the LPS is administered in vivo. When LPS is used to induce TNF in whole blood, in vitro administration of an H_4_R antagonist does not inhibit this response. This suggests that cells in the blood are not the source of TNF in vivo even though they can produce TNF in vitro. Analysis of TNF RNA levels showed that LPS increased the TNF message in the liver to a greater extent than in blood thereby supporting the hypothesis that tissue resident cells are the source of LPS-induced TNF. Since H_4_R antagonism only inhibits TNF production by LPS in vivo, it is possible that the receptor is only active on tissue resident macrophages. The TNF immunofluorescence can be detected in the liver after LPS administration and this co-stains with a marker of macrophages. H_4_R expression can also be detected in Kupffer cells albeit at low levels. As further confirmation of this we have shown that inhibiting or depleting tissue macrophages blocked the production of TNF induced by LPS.

The in vivo only inhibitory activity of H_4_R antagonist on LPS-induced TNF production is in contrast to other inhibitors of LPS-induced TNF production like p38 kinase inhibitors, which have effects in vivo and in vitro [15]. The difference between resident cells and those derived from blood has been seen previously with H_4_R effects on dendritic cells. The H_4_R could mediate TLR driven cytokine responses in mouse splenic CD11c^+^ cells [2], but not from mouse monocyte-derived dendritic cells (O’Donnell and Thurmond, unpublished data). The involvement of H_4_R in TNF production in the liver may play a role in liver injury. The TNF has been shown to be a major mediator of GalN/LPS induced liver injury [22] and here we show that an H_4_R antagonist can inhibit the induction of ALT in this model.

Currently, the mechanism by which the H_4_R modulates LPS-induced TNF production is not known and unraveling these mechanisms is complicated since the effect only occurs in vivo. The impact on NFκB and AP-1 activation in the liver was studied and, although their activation was increased by LPS, this was not modulated by antagonism of the H_4_R (data not shown). However, previously it was shown that in mouse mast cells there is synergy between H_4_R and TLR activation in the production of IL-6 [11]. In this case, both H_4_R and TLR activation led to activation of MAP kinases and phosphoinositide 3-kinase gamma, however, the combination of both signals did not increase the levels of kinase activation, but instead led to sustained activation. This was hypothesized to underlie the increased IL-6 production in mast cells and may also impact the TNF production seen in vivo. However, further work is necessary to completely understand the details of this interaction.

The impact of the H_4_R on TLR-induced cytokine responses both in vivo and in vitro provides a basis for understanding the anti-inflammatory properties of H_4_R antagonists in various animal models. Previously, we have shown that the H_4_R can modulate inflammatory responses in a mouse asthma model and it has been reported that LPS is necessary for obtaining a robust inflammatory response in these models [23, 24]. We confirm both of these findings here and show that LPS was also necessary to evoke an H_4_R component of the inflammation. Therefore, the anti-inflammatory action of an H_4_R antagonist as previously shown is dependent on the presence of LPS. It is possible that TLR activation or other similar signals are required for the activation of dendritic cells and macrophages and that these in turn drive T cell-dependent aspects of the model. Activation of the H_4_R is needed to amplify TLR responses on these cells. This interaction may also underlie other models such as those for colitis. The H_4_R antagonists block the inflammation in a rat model of colitis [25]. In this model chemical damage to the colon exposes submucosal tissue to bacteria that activate TLR signaling and thereby drive the inflammatory response. The anti-inflammatory effects of H_4_R antagonist in this model may be due to inhibition of the TLR-driven response. Indeed in the colitis model, treatment with the H_4_R antagonists completely inhibited the increase in tissue TNF levels [25].

In conclusion, antagonism of the H_4_R can inhibit TLR-driven inflammatory responses in vivo. This, along with previously published in vitro data, provides evidence for an interaction between activation of the H_4_R and activation of TLR that drives inflammatory responses. These findings may have an impact in human diseases since both TLR and the H_4_R have been implicated in conditions such as asthma, autoimmune diseases and atopic dermatitis.

## Electronic supplementary material

Below is the link to the electronic supplementary material.

Supplementary material 1 (PDF 840 kb)

## Abbreviations

H_4_R: Histamine H_4_ Receptor
MP: Methylpalmitate
GaIN: Galactosamine
CL: Clodronate liposomes
ALT: Alanine transaminase
