# Supplementary material for: Antagonism of the histamine H4 receptor reduces LPS-induced TNF production in vivo
Source: Inflamm Res. 2013 Mar 27;62(6):599–607. doi: 10.1007/s00011-013-0612-5 (PMC3654183; doi:10.1007/s00011-013-0612-5)
Supplement: Supplementary file 1 — Supplementary material 1 (PDF 840 kb) [file 11_2013_612_MOESM1_ESM.pdf]

SUPPLEMENTAL DATA

Table S1: Pharmacokinetics of JNJ 28307474 in mice. Single dose pharmacokinetics in mice of JNJ 28307474 was been evaluated in non-fasted BALB/c female mice. Compound was administered orally (in 20% hydroxypropyl-beta-cyclodextrin). Three mice were used per time point. LC/MS analysis was used to quantitate the plasma levels.

Dose	T_{max} (h)	C_{max} (μM)	AUC (h*μM)	$t_{1/2}$ (h)
3 mg/kg	1.00	0.33	4.02	5.03
20 mg/kg	1.00	3.76	32.59	4.30

Figure S1. Inactivation of liver macrophages by MP. Female wild-type mice (7-8 mice per group) were treated with 2 g/kg MP or vehicle by tail vein injection. Forty-four hours later, the livers were collected and stained with F4/80 to visualize the number of macrophages.

Figure S2. Depletion of liver macrophages by CL. A) Female wild-type mice (7-8 mice per group) were treated with 10 mg/kg CL or vehicle (empty liposomes; EL) by tail vein injection 44 h before administration of 20 µg LPS or vehicle i.p. Two hours later, the livers were collected and stained with F4/80 to quantitate the number of macrophages (A). The number of F4/80 positive cells was quantitated and the data presented as the average along with SEM (B).

Figure S3. LPS induces TNF production from F4/80$^+$ cells in the liver. Livers were collected from untreated mice (A), mice treated with 20 µg LPS i.p. (B), or mice treated with with 20 µg ng/mL LPS i.p. and 20 mg/kg JNJ 7777120 (C). Frozen sections were stained with F4/80 (green) and anti-TNF (red) and detected by immunofluoresence.

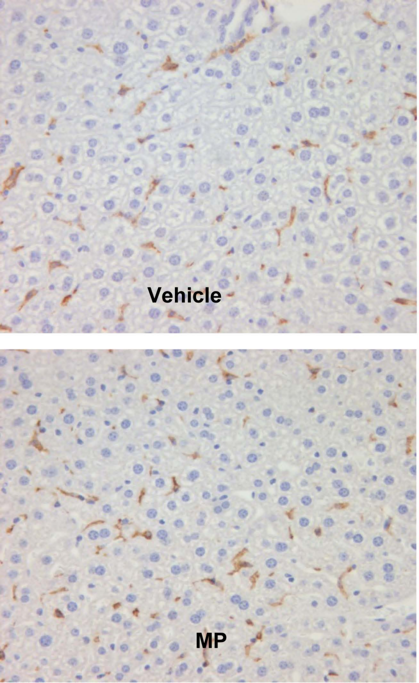
Vehicle
MP

A)

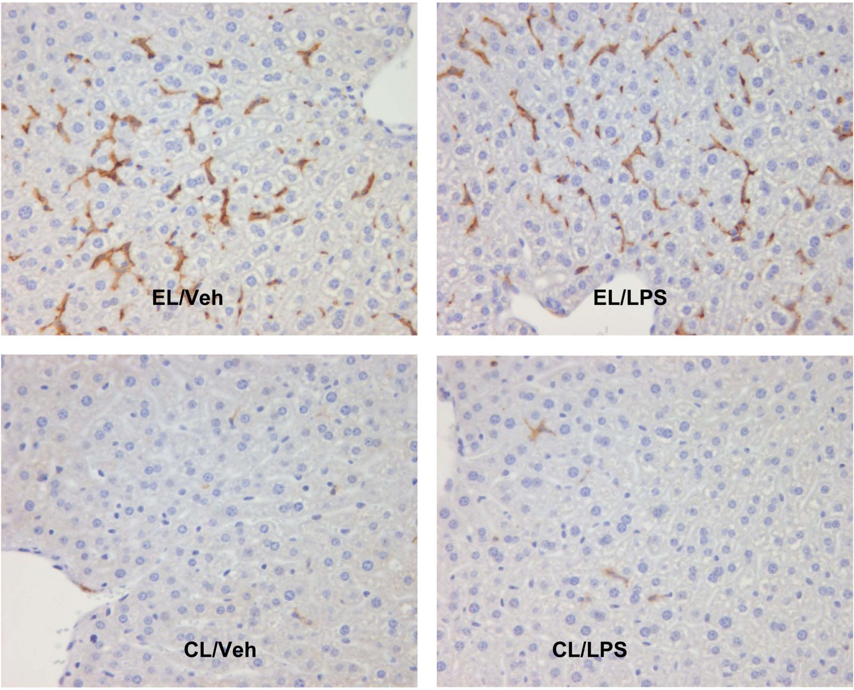
EL/Veh
EL/LPS
CL/Veh
CL/LPS

B)

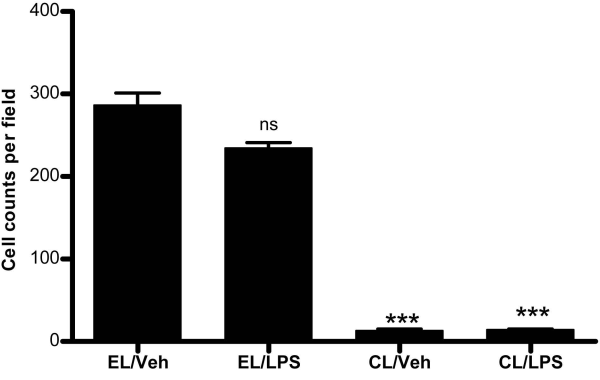
Cell counts per field
400
300
200
100
0
ns
***
***
EL/Veh
EL/LPS
CL/Veh
CL/LPS

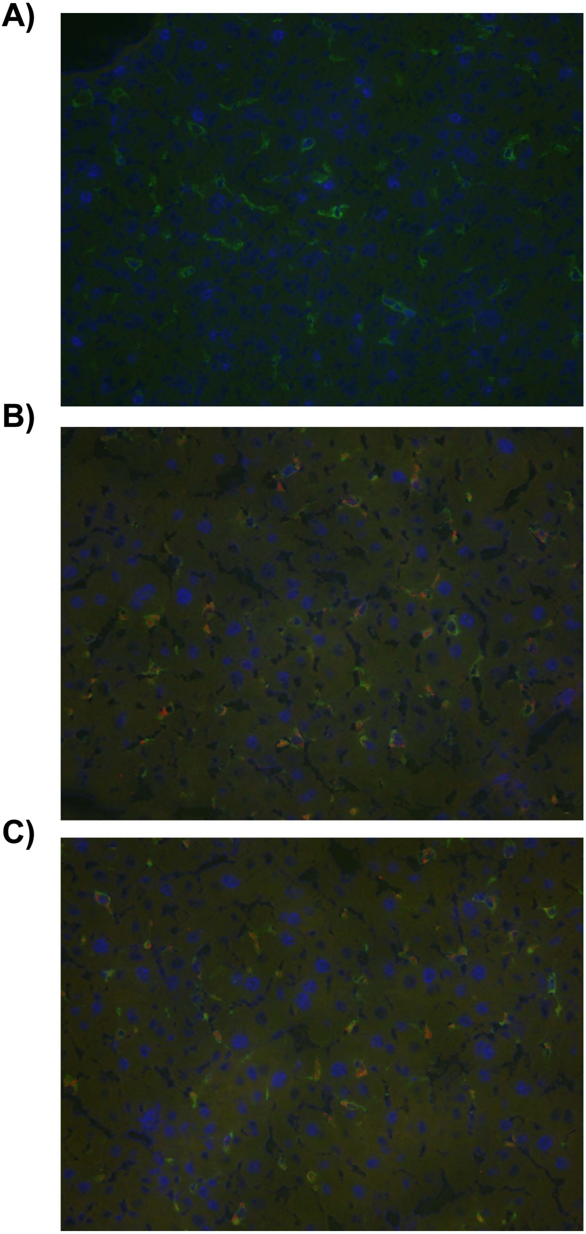

Figure S3
